# TMEM59 interacts with TREM2 and modulates TREM2-dependent microglial activities

**DOI:** 10.1038/s41419-020-02874-3

**Published:** 2020-08-13

**Authors:** Zhaoji Liu, Jinhuan Ning, Xiaoyuan Zheng, Jian Meng, Linkun Han, Honghua Zheng, Li Zhong, Xiao-Fen Chen, Xian Zhang, Hong Luo, Dan Can, Huaxi Xu, Yun-wu Zhang

**Affiliations:** 1 Department of Neurology, Zhongshan Hospital Xiamen University, 361004 Xiamen Fujian, China; 2 Fujian Provincial Key Laboratory of Neurodegenerative Disease and Aging Research, Institute of Neuroscience, School of Medicine, Xiamen University, 361102 Xiamen Fujian, China; 3 Department of Neurology, The First Affiliated Hospital of Xiamen University, 361003 Xiamen Fujian, China

**Keywords:** Cellular neuroscience, Alzheimer's disease, Microglia, Homeostasis

## Abstract

The surface receptor triggering receptor expressed on myeloid cells 2 (TREM2) plays a crucial role in maintaining a multitude of microglial activities, such as survival, proliferation, migration, metabolism, inflammation, and phagocytosis. However, the molecular mechanisms underlying TREM2-mediated microglial activities remain largely elusive. Herein, we found that TREM2 interacted with the type I transmembrane protein TMEM59, whose expression could facilitate autophagic flux through its carboxyl-terminus. TMEM59 expression was decreased upon lipopolysaccharide treatment. While downregulation of TMEM59 promoted anti-inflammatory factor expression and attenuated lipopolysaccharide treatment-induced inflammation. Importantly, we found that overexpression of TREM2 reduced TMEM59 protein levels through promoting its degradation, whereas TMEM59 levels were elevated in *Trem2-*deficient microglia. Finally, impaired survival, proliferation, migration, and phagocytosis, as well as dysregulated autophagy and metabolism in *Trem2*-deficient microglia were attenuated upon TMEM59 silencing. Together, our findings reveal a novel function of TREM2 in mediating TMEM59 protein degradation and demonstrate the importance of TMEM59 homeostasis in maintaining TREM2-mediated microglial activities.

## Introduction

Microglia are the principal immune cells residing in the central nervous system (CNS) and crucial for brain immunosurveillance^1^. In addition to functioning in host defense against infectious pathogens, microglia have been found to participate in various neurological disorders including neurodevelopment and neurodegenerative diseases^2–5^. Alzheimer’s disease (AD) is the most common neurodegenerative disease and multiple genes important for maintaining normal microglia functions, such as triggering receptor expressed on myeloid cells 2 (*TREM2*), apolipoprotein E (*APOE*), complement receptor 1 (*CR1*), cluster of differentiation 33 (*CD33*), and ATP-binding cassette transporter A7 (*ABCA7*) have been genetically linked to AD^6^.

TREM2 is a type 1 transmembrane receptor protein containing an extracellular domain, a transmembrane domain, and a short cytoplasmic tail^7^. In the CNS, TREM2 is dominantly expressed in microglia and interacts with the protein adaptor DAP12, which transmits intracellular signals upon ligand binding of TREM2^8^. It has been reported that the extracellular domain of TREM2 can bind to bacterial lipopolysaccharide (LPS)^9^, phospholipids^10^, amyloid-β (Aβ) oligomers^11–13^, APOE and APOJ and related lipoproteins^14–16^, and apoptotic neurons^17^. Activation of the TREM2-DAP12-signaling pathway promotes microglial survival, proliferation, clustering, and phagocytosis^18–21^. Homozygous loss-of-function mutations in *TREM2* as well as *DAP12* have been reported to cause Nasu–Hakola disease (NHD), in which patients develop systemic bone cysts and presenile dementia^22,23^. More recently, heterozygous *TREM2* mutations have been found to be associated with AD^24–26^, and then with Parkinson’s disease, frontotemporal dementia, and amyotrophic lateral sclerosis^27–30^. AD-associated mutations lead to TREM2 dysfunction, so that microgliosis is compromised in such mutation-carrying patients and related AD model mice^31,32^. Both increased and decreased DNA methylation in the *TREM2* gene have been reported^33–36^, though increased TREM2 expression has been consistently found in AD patients and mouse models and may be associated with the recruitment of microglia to amyloid plaques^37^. Moreover, TREM2 deficiency has been found to impair mTOR activation, increase autophagy, and cause metabolic dysfunctions in microglia^18,20,38^. Since dysregulated autophagy also plays an important role in AD^39,40^, elucidation of the molecular mechanism underlying TREM2 deficiency-caused autophagy change may provide new insight into disease intervention.

TMEM59 (also known as dendritic cell-derived factor 1, DCF1) is a ubiquitously expressed type I transmembrane protein. TMEM59 contains an ATG16L1-binding motif that promotes local activation of LC3 and evokes autophagy^41,42^. Previous studies suggested that TMEM59 could regulate neural stem cell differentiation^43,44^, dendritic spine development^45^, neuropeptide expression^46^, glioblastoma cell proliferation/apoptosis^47,48^, the Wnt signaling through interacting with the Wnt receptor Frizzled (FZD)^49^, and glycosylation and processing of the Alzheimer-associated Aβ precursor protein^50^. Interestingly, hypomethylation of the *TMEM59* gene has been found in postmortem frontal cortex of late-onset AD patients^51^, suggesting that TMEM59 may be affected by AD and possibly other neurological or neurodegenerative diseases. One recent work noted that TMEM59 deficiency attenuated microglia activation during neuroinflammation, but the underlying mechanism was unknown^52^. Herein, we further confirmed that TMEM59 could facilitate autophagic flux through its carboxyl-terminus. Moreover, we found that LPS treatment reduced TMEM59 expression, whereas downregulation of TMEM59 promoted anti-inflammatory factor expression and alleviated LPS treatment-induced neuroinflammation. Importantly, we demonstrated that TMEM59 could interact with TREM2, which in turn mediated the degradation of TMEM59. While downregulation of TMEM59 promoted microglial survival, proliferation, migration, phagocytosis, and mitochondrial function, and attenuated impairments of these activities in TREM2-deficient microglia.

## Results

### TMEM59 promotes autophagy through its carboxyl terminus

**Fig. 1:**
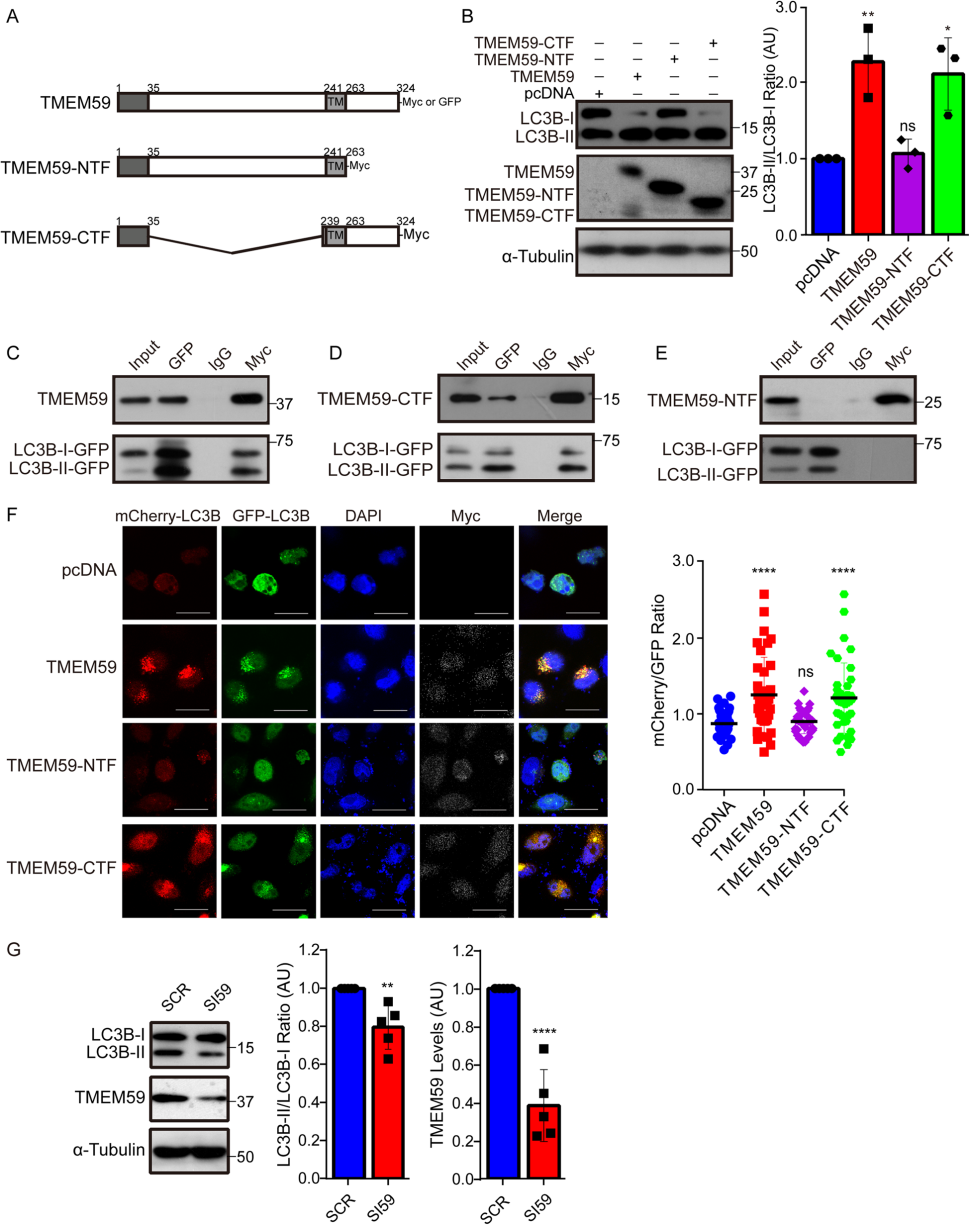
TMEM59 promotes autophagy flux through its CTF. **a** Schemes of TMEM59 and its truncated constructs. These constructs were tagged with Myc or GFP on the C-termini. TM: transmembrane domain. **b** BV2 cells were transfected with control pcDNA or Myc-tagged TMEM59, TMEM59-NTF, and TMEM59-CTF for 24 h. Equal protein amounts of cell lysates were immunoblotted for proteins indicated. Protein levels were quantified by densitometry. The ratio of LC3B-II/LC3B-I was calculated and compared to controls (set to one arbitrary units, AU). Data represent mean ± SEM (*n* = 3). **c–e** The mCherry-GFP-LC3B plasmid was co-transfected with Myc-tagged TMEM59 **c**, TMEM59-CTF **d**, or TMEM59-NTF **e** into BV2 cells for 24 h. Cell lysates were subjected to immunoprecipitation with antibodies against Myc or GFP and control mouse immunoglobulin G (IgG), and then immunoblotted with antibodies against Myc or GFP to recognize various TMEM59 forms or LC3B. **f** HEK293T cells were co-transfected with mCherry-GFP-LC3B and various TMEM59 truncated plasmids for 24 h. Cells were immunostained with an anti-Myc antibody (white) and counterstained with DAPI (blue), and then observed under a confocal microscope. Red and green colors indicate mCherry and GFP, respectively. Red and green color intensities in cells with positive Myc immunostaining were quantified by ImageJ, and the ratio of mCherry/GFP signals was calculated and compared. Scale bars: 25 μm. Data represent mean ± SEM (*n* = 36 cells from four experiments). **g** BV2 cells were transfected with a scramble siRNA (SCR) or *Tmem59* siRNA (SI59) for 48–72 h. Equal protein amounts of cell lysates were immunoblotted for indicated proteins. Protein levels were quantified by densitometry and compared to respective controls (set to one AU). Data represent mean ± SEM (*n* = 5). ns, *p* > 0.05, *p < 0.05, **p < 0.01, ****p < 0.0001 (one-way ANOVA with Tukey’s post hoc test for **b** and **f**, and Mann–Whitney *U* test for **g)**.

Our previous work as well as others have shown that overexpression of full-length TMEM59 could induce LC3 activation. One study suggested that an intracellular domain of TMEM59 mediated its interaction with ATG16L1 for this activity^41^. Here we generated human TMEM59 amino-terminal fragment (NTF) and carboxyl-terminal fragment (CTF) (Fig. 1a) plasmids and found that overexpression of TMEM59-CTF, but not TMEM59-NTF, could dramatically increase the ratio of LC3B-II/LC3B-I just like overexpression of full length TMEM59 did in both HEK293T cells (Supplementary Fig. 1) and the mouse microglial BV2 cell line (Fig. 1b). When different TMEM59 plasmids were co-transfected with a GFP-tagged LC3B plasmid into BV2 cells, we found that both full length TMEM59 (Fig. 1c) and TMEM59-CTF (Fig. 1d) interacted with LC3B, whereas TMEM59-NTF did not (Fig. 1e). To further study whether TMEM59 overexpression promotes the ratio of LC3B-II/LC3B-I through enhancing autophagic flux or through interfering with the autolysosome formation and thus LC3B-II degradation, we co-expressed different TMEM59 plasmids with an mCherry-GFP-LC3B plasmid in HEK293T cells. As the GFP signal is quenched under an acidic environment in the autophagolysosome, an increase of the mCherry/GFP signal ratio may indicate elevated autophagic flux. We found that overexpression of both full length TMEM59 and TMEM59-CTF, but not TMEM59-NTF significantly increased the ratio of mCherry/GFP signal (Fig. 1f). Moreover, downregulation of TMEM59 reduced the ratio of LC3B-II/LC3B-I in BV2 cells (Fig. 1g). Together, our results suggest that TMEM59 promotes autophagy through its CTF.

### TMEM59 is involved in neuroinflammation

**Fig. 2:**
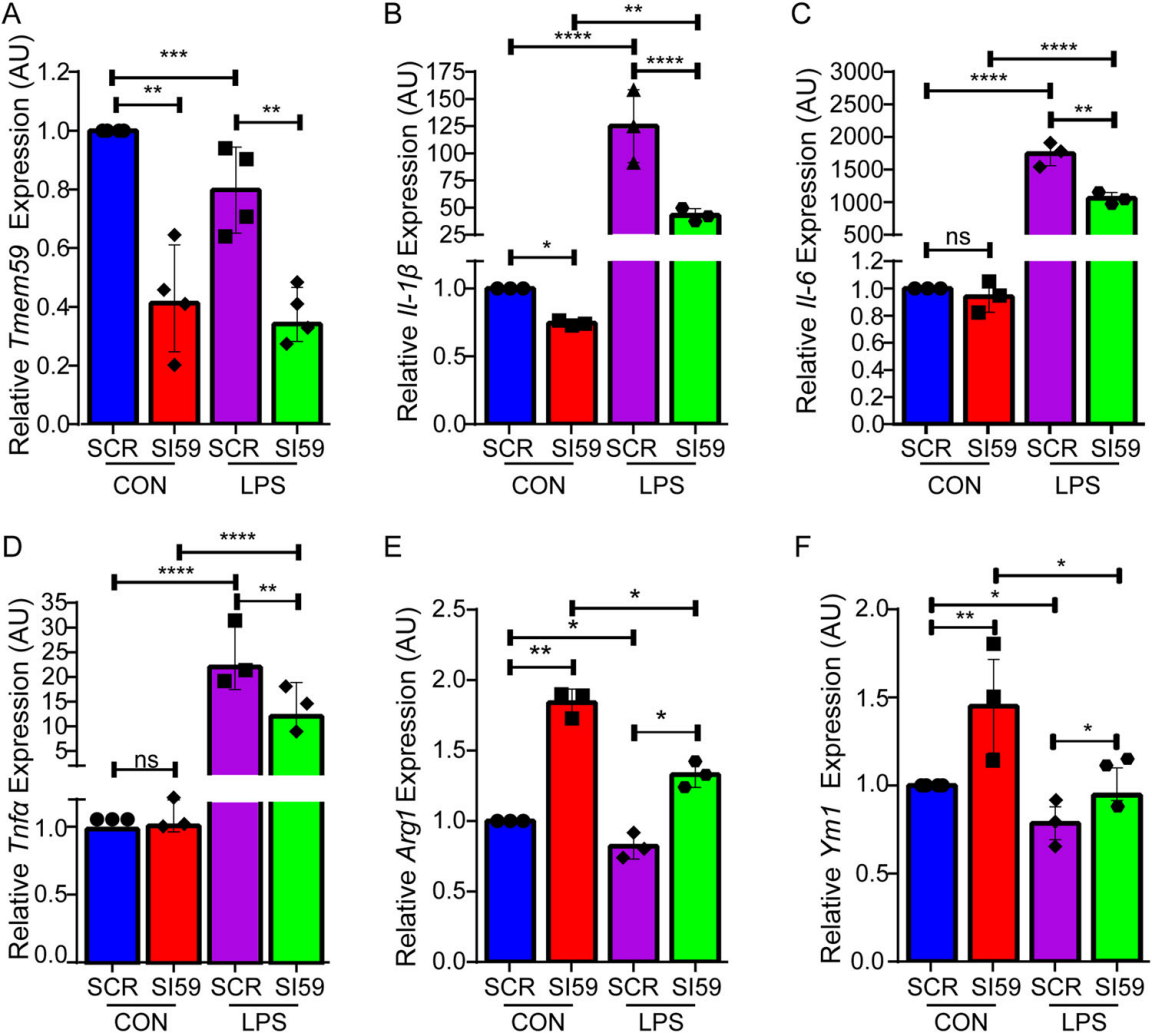
TMEM59 is involved in inflammation in microglia. **a–f** Mouse primary microglia were transfected with a scramble control (SCR) or *Tmem59* siRNA (SI59) for 48 h, and then treated with 500 ng/ml LPS or vehicle control (CON) for 6 h. Gene expression levels of *Tmem59* **a**, *Il-1β* **b**, *Il-6* **c**, *Tnfα* **d**, *Arg1* **e**, and *Ym1* **f** were determined by qRT-PCR and compared to respective controls (set to one arbitrary units, AU). Data represent mean ± SEM (*n* = 3 or 4). ns, *p* > 0.05, *p < 0.05, **p < 0.01, ***p < 0.001, ****p < 0.0001 (one-way ANOVA with Tukey’s post hoc test).

As microglia are the primary immune effector cells in the CNS, we studied the potential role of TMEM59 in neuroinflammation. Delivery of a *Tmem59*-specific siRNA into mouse primary microglia significantly reduced TMEM59 protein levels at 48–72 h after electroporation (Supplementary Fig. 2). Moreover, when *Tmem59* expression was downregulated in mouse primary microglia (Fig. 2a) and BV2 cells (Supplementary Fig. 3a) by siRNA, the expression of the pro-inflammatory factor IL-1β (Fig. 2b and Supplementary Fig. 3b), but not another two pro-inflammatory factors IL-6 and TNFα (Fig. 2c, d and Supplementary Fig. 3c, d), was significantly reduced. While the expression of anti-inflammatory factors including ARG1 and YM1 was significantly increased (Fig. 2e, f and Supplementary Fig. 3e, f). Interestingly, when cells were treated with LPS, *Tmem59* expression was markedly reduced (Fig. 2a and Supplementary Fig. 3a). Moreover, downregulation of *Tmem59* attenuated the elevated expression of pro-inflammatory factors and decreased the expression of anti-inflammatory factors upon LPS treatment (Fig. 2b–f, and Supplementary Fig. 3b–f). Overall, the results from primary microglia and BV2 cells were comparable, except that LPS treatment had a better effect on reversing *Tmem59* downregulation-induced *Ym1* expression in primary microglia (about 34% reduction, Fig. 2f) than in BV2 cells (about 10% reduction, Supplementary Fig. 2f). Together, our results suggest that TMEM59 is involved in the microglial inflammatory activity and that downregulation of TMEM59 can ameliorate LPS treatment-induced inflammation.

### TMEM59 interacts with TREM2

**Fig. 3:**
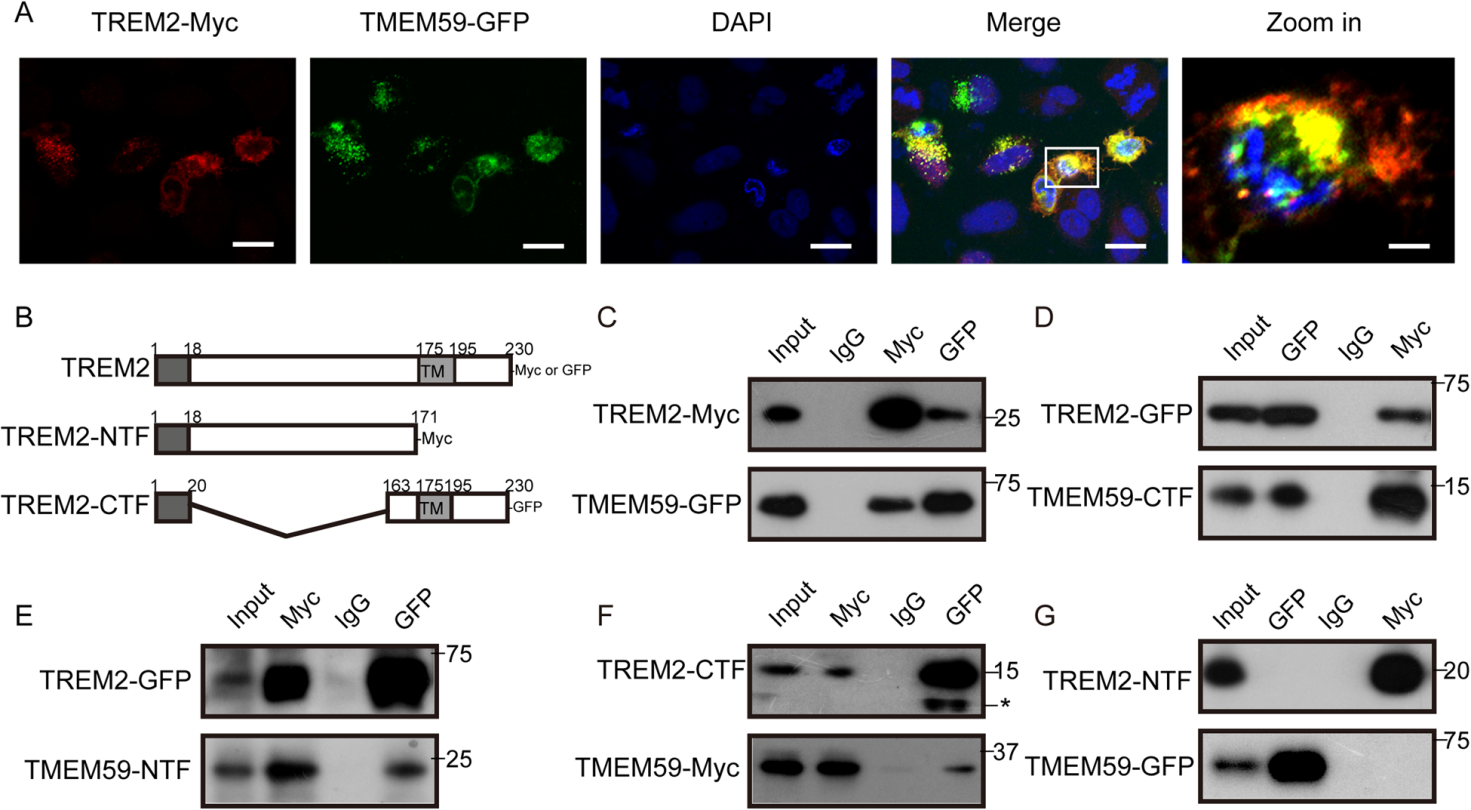
TREM2 interacts with TMEM59. **a** Hela cells were transfected with TREM2-Myc and TMEM59-GFP for 24 h. Cells were immunostained with an antibody against Myc (for TREM2, red) and counterstained with DAPI (blue), and observed under a confocal microscope. TMEM59-GFP is shown in green. Scale bars: 45 μm for the first four images and 15 μm for the “zoom in” image. **b** Schemes of TREM2 and its truncated constructs. These constructs were tagged with Myc or GFP on the C-termini. TM: transmembrane domain. **c–g** BV2 cells were co-transfected with TREM2-Myc and TMEM59-GFP **c**, TREM2-GFP and TMEM59-CTF-Myc **d**, TREM2-GFP and TMEM59-NTF-Myc **e**, TREM2-CTF-GFP and TMEM59-Myc **f**, or TREM2-NTF-Myc and TMEM59-GFP **g** for 24 h. Cell lysates were immunoprecipitated with antibodies against GFP and Myc and mouse immunoglobulin G (IgG), and immunoblotted for the components indicated. Ten percent amounts of cell lysates for immunoprecipitation were used as input. *Non-specific band.

Since both TREM2 and TMEM59 are type I transmembrane proteins, we explored their potential interactions. We found marked colocalizations between co-expressed TREM2 and TMEM59 (Fig. 3a). We generated various TREM2 and TMEM59 truncated constructs (Figs. 1a and 3b) and carried out co-immunoprecipitation assays in BV2 cells. We indeed found that full-length TREM2 interacted with full-length TMEM59 (Fig. 3c). Similar to TREM2, the receptor for colony-stimulating factor-1 (CSF1R) is also a type I transmembrane protein and expressed in microglia, and plays an essential role for microglial survival and physiological functions^53^. Therefore, we used CSF1R as a control and found that TMEM59 had no interaction with CSF1R (Supplementary Fig. 4), suggesting that the TMEM59–TREM2 interaction is specific.

Moreover, we found that full-length TREM2 interacted with TMEM59-CTF (Fig. 3d) and TMEM59-NTF (Fig. 3e), both of which possess the transmembrane domain. While full-length TMEM59 interacted with TREM2-CTF that contains the transmembrane domain (Fig. 3f) but not TREM2-NTF that has no the transmembrane domain (Fig. 3g). Together, these results indicate that TREM2 interacts with TMEM59 most likely through their transmembrane domains.

### TREM2 promotes TMEM59 protein degradation

**Fig. 4:**
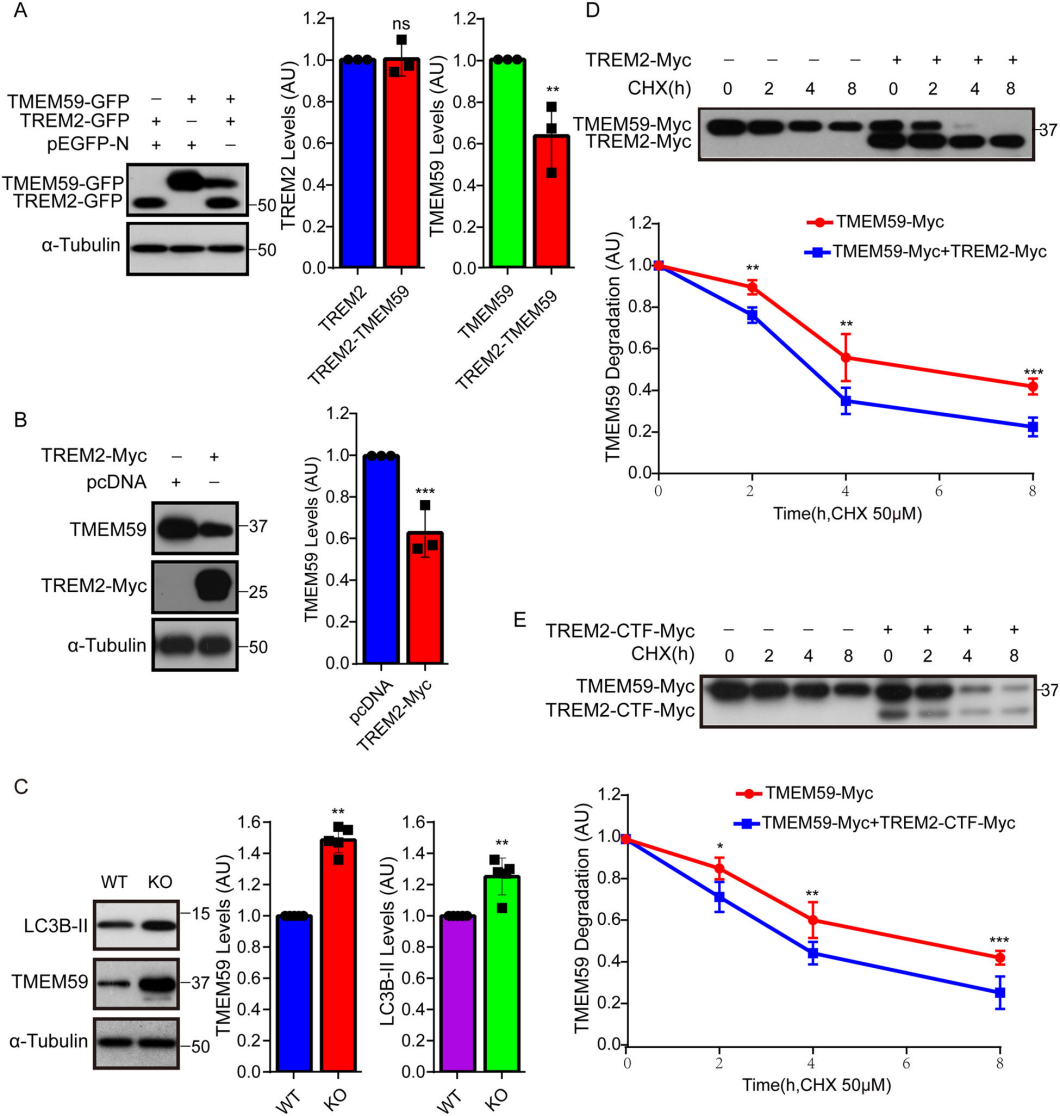
TREM2 promotes TMEM59 protein degradation. **a** BV2 cells were transfected with pEGFP-N control plus TREM2-GFP, pEGFP-N plus TMEM59-GFP, or TMEM59-GFP plus TREM2-GFP for 24 h. Equal protein amounts of cell lysates were immunoblotted for proteins indicated. Protein levels were quantified by densitometry and compared to respective controls (set to one arbitrary units, AU). Data represent mean ± SEM (*n* = 3). **b** BV2 cells were transfected with pcDNA or TREM2-Myc for 24 h. Equal protein amounts of cell lysates were subjected to immunoblotting. TMEM59 protein levels were quantified by densitometry and compared to controls (set to one AU). Data represent mean ± SEM (*n* = 3). **c** Primary microglia derived from wild type (WT) and *Trem2* KO mice were lysed. Equal protein amounts of cell lysates were immunoblotted for proteins indicated. Protein levels were quantified and compared to controls (set to one AU). Data represent mean ± SEM (*n* = 5). **d**, **e** In BV2 cells, TMEM59-Myc was co-transfected with control (−) or TREM2-Myc (+, **d**), or TREM2-CTF-Myc (+, **e**). Cells were then equally split, and treated with 50 μM cycloheximide (CHX) for the time indicated. TMEM59 and TREM2 proteins were subjected to immunoblotting with an anti-Myc antibody. TMEM59-Myc protein levels were quantified by densitometry for comparison (values at the 0 h time point were set to one AU). Data represent mean ± SEM (*n* = 3). ns, *p* > 0.05, *p < 0.05, **p < 0.01, ***p < 0.001 (Mann–Whitney *U* test).

When TREM2 was co-expressed with TMEM59 in BV2 cells, we noticed that TMEM59 protein levels were dramatically reduced compared to controls, whereas co-expression of TMEM59 did not affect TREM2 protein levels (Fig. 4a). Overexpression of TREM2 also reduced endogenous TMEM59 protein levels in BV2 cells (Fig. 4b). However, overexpression of TREM2 had no effect on endogenous *Tmem59* mRNA levels (Supplementary Fig. 5a), and overexpression of TMEM59 had no effect on endogenous *Trem2* mRNA levels in BV2 cells (Supplementary Fig. 5b). Moreover, TMEM59 protein levels were significantly increased in *Trem2* knockout (KO) microglia, accompanied by increased LC3B-II levels (Fig. 4c). While *Tmem59* mRNA expression was not altered in *Trem2* KO microglia (Supplementary Fig. 5c). Downregulation of TMEM59 had no effect on endogenous *Trem2* mRNA levels in mouse primary microglia (Supplementary Fig. 5d). These results suggest that TREM2 affects TMEM59 protein levels at post-translational levels.

When cycloheximide (CHX) was applied to inhibit protein synthesis in BV2 cells, we found that co-expressing full length TREM2 (Fig. 4d) and TREM2-CTF (Fig. 4e) but not TREM2-NTF (Supplementary Fig. 5e) dramatically promoted TMEM59 protein degradation, whereas co-expressing TMEM59 had no effect on TREM2 degradation (Supplementary Fig. 5f). Co-expressing TREM2-CTF (Supplementary Fig. 5g) but not TREM2-NTF (Supplementary Fig. 5h) also significantly reduced TMEM59 levels. Furthermore, both co-treatment with the proteasome inhibitor MG132 and with the lysosome inhibitor NH_4_Cl rescued the degradation of TMEM59 (Supplementary Fig. 5i), suggesting that TMEM59 is degraded through both proteasomal and lysosomal pathways. However, co-treatment with MG132 or NH_4_Cl showed no effect on reversing full-length TREM2 degradation (Supplementary Fig. 5i). One possible explanation for this is that during its degradation, TREM2 can still be cleaved by α- and γ-secretases^54,55^, so that inhibition of proteasomal or lysosomal pathways fails to restore full-length TREM2 levels.

### Downregulation of TMEM59 promotes microglial activities and attenuates microglial activity impairment in *Trem2* KO microglia

**Fig. 5:**
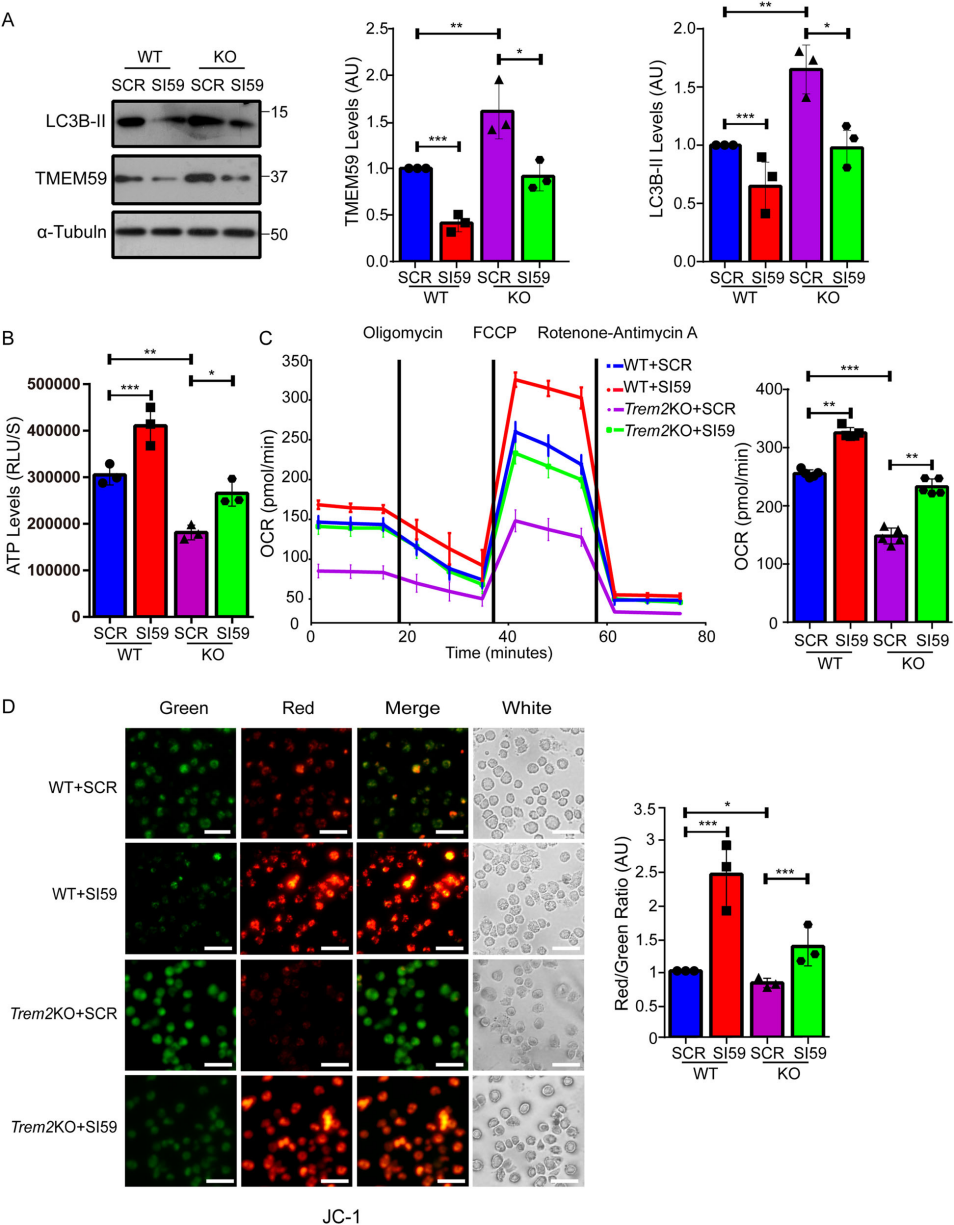
TMEM59 downregulation attenuates elevated autophagy and impaired mitochondrial functions in *Trem2* KO microglia. Primary microglia derived from wild type (WT) and *Trem2* KO mice were transfected with a scramble control (SCR) or *Tmem59* siRNA (SI59) for 48–72 h. **a** Cells were lysed, and equal protein amounts of cell lysates were immunoblotted for proteins indicated. Protein levels were quantified by densitometry and compared to controls (WT microglia transfected with SCR, set to one arbitrary units, AU). Data represent mean ± SEM (*n* = 3). **b** Cells were measured for ATP levels for comparison. Data represent mean ± SEM (*n* = 3). **c** Cells were assayed for baseline oxygen consumption rate (OCR) using a Seahorse analyzer. Data represent mean ± SEM (*n* = 5). **d** Cells were subjected to JC-1 staining to measure mitochondrial membrane potential. The fluorescence intensities of JC‑1 monomers that dominate in depolarized mitochondria (in green) and JC-1 aggregates that dominate in polarized mitochondria (in red) were visualized under a confocal microscope. Red and green color intensities were quantified by ImageJ, and the ratio of red/green signal was calculated and compared. Data represent mean ± SEM (*n* = 3). Scale bars: 45 μm. *p < 0.05, **p < 0.01, ***p < 0.001 (one-way ANOVA with Tukey’s post hoc test).

TREM2 plays an essential role in maintaining normal microglial activities. TREM2 deficiency can alter autophagy and cause mitochondria dysfunctions in microglia^18,20,38^. Therefore, we studied whether increased TMEM59 in *Trem2* KO microglia mediates these impairments. We found that downregulation of TMEM59 not only reduced LC3B-II levels in wild type (WT) microglia, but also reversed the elevation of LC3B-II levels in *Trem2* KO microglia (Fig. 5a). Moreover, we noticed that downregulation of TMEM59 promoted ATP production and oxygen consumption rate (OCR) in WT microglia, and rescued the compromised ATP production and OCR in *Trem2* KO microglia (Fig. 5b, c). Mitochondrial membrane potential is related to the capacity for cells to generate ATP by oxidative phosphorylation and has been considered as a key indicator of cell health or injury^56^. We also found that mitochondrial membrane potential was impaired in *Trem2* KO microglia, and downregulation of TMEM59 dramatically increased mitochondrial membrane potential in both WT and *Trem2* KO microglia (Fig. 5d).

**Fig. 6:**
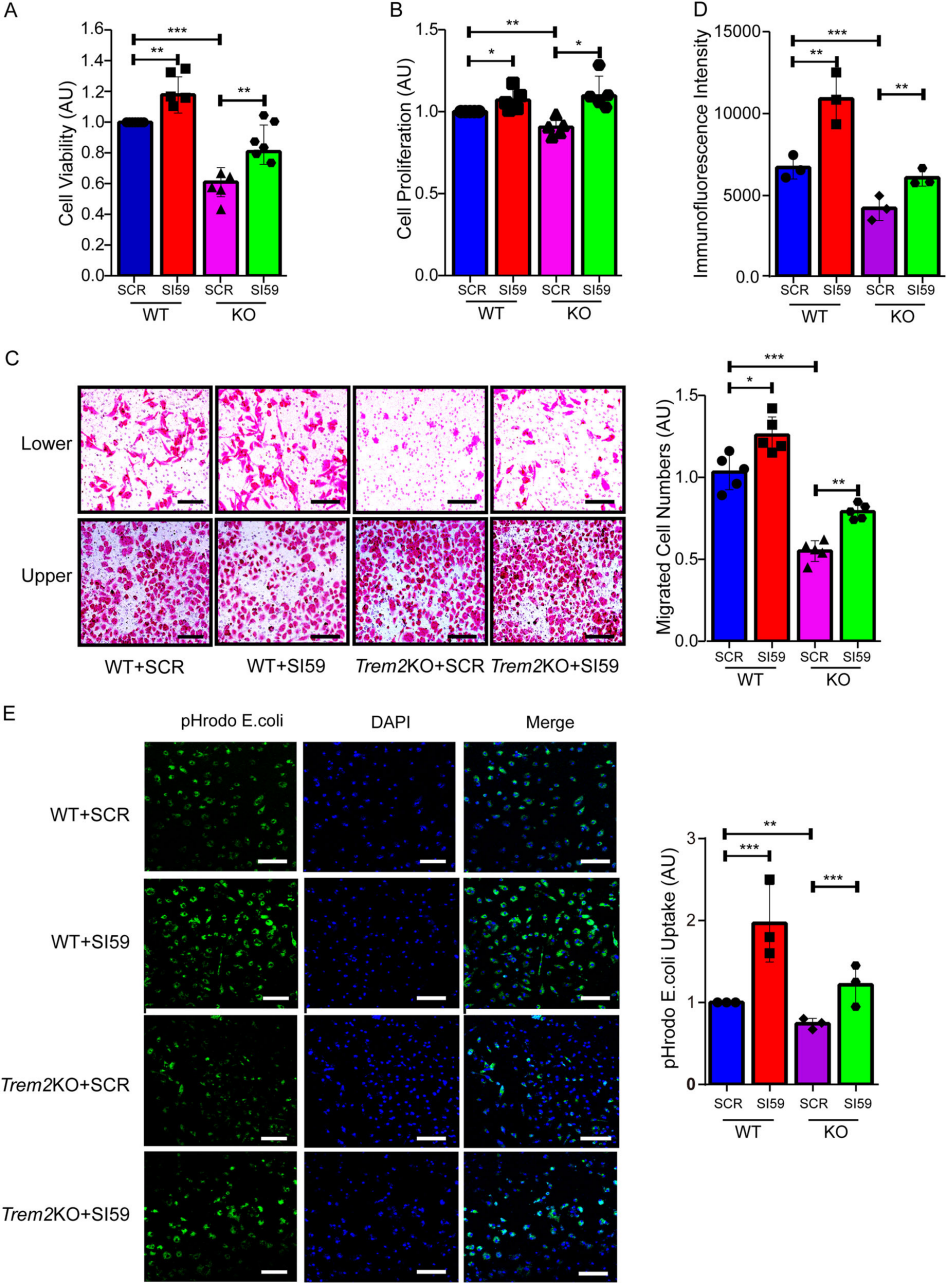
TMEM59 downregulation ameliorates impaired survival, proliferation, migration, and phagocytosis in *Trem2* KO microglia. **a–e** Primary microglia derived from wild type (WT) and *Trem2* KO mice were transfected with a scramble control (SCR) or *Tmem59* siRNA (SI59) for 48–72 h. Cell viability was studied using the CCK8 assay **a**. Data represent mean ± SEM (*n* = 6). Cell proliferation was assessed by BrdU incorporation **b**. Data represent mean ± SEM (*n* = 5). Cell migration was determined by transwell assays **c**. Data represent mean ± SEM (*n* = 5). Scale bars: 45 μm. Phagocytosis of the pH-dependent pHrodo *E. coli* BioParticles was analyzed by FACS **d** and fluorescent microscopy **e**. Data represent mean ± SEM (*n* = 3). Scale bars: 20 μm. ns *p* > 0.05, *p < 0.05, **p < 0.01, ***p < 0.001 (one-way ANOVA with Tukey’s post hoc test).

Previous studies have suggested that TREM2 deficiency impairs various microglial activities^18–21^. Here we confirmed that *Trem2* KO microglia had deficits in survival (Fig. 6a), proliferation (Fig. 6b), migration (Fig. 6c), and phagocytotic activity (Fig. 6d, e). Moreover, we found that downregulation of TMEM59 promoted all these microglial activities in WT microglia and attenuated all observed impairments in *Trem2* KO microglia (Fig. 6a–e). Finally, we found that when WT microglia were subjected to deprivation of the granulocyte macrophage colony stimulating factor (GM-CSF) (Supplementary Fig. 6a, b) or endoplasmic reticulum (ER) stress induced by tunicmycin treatment (Supplementary Fig. 6c, d), downregulation of TMEM59 could also promote cell survival and proliferation, implying that downregulation of TMEM59 exerts a broad protection against various cell stresses.

## Discussion

TREM2 is specifically expressed in microglia in the CNS and plays an essential role in maintaining normal microglial activities. *TREM2* mutations have recently been associated with AD and some other neurodegenerative diseases. However, the detailed molecular mechanisms underlying the participation of TREM2 in various physiological and pathological conditions have yet to be fully elucidated.

**Fig. 7:**
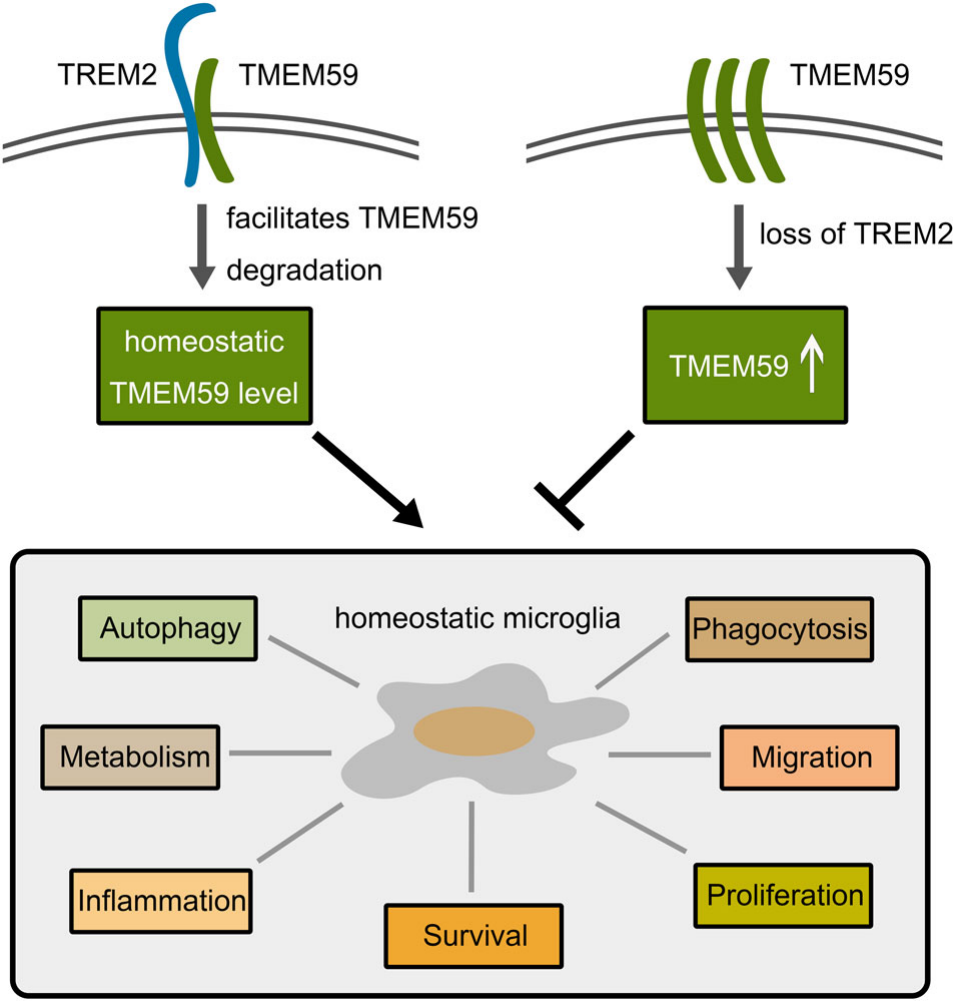
The schematic diagram showing that TMEM59 homeostasis regulated by TREM2 maintains physiological microglial activities. Under normal conditions, TREM2 interacts with TMEM59 most likely through their transmembrane domains and facilitates TMEM59 protein degradation. Homeostatic TMEM59 levels maintain physiological microglial activities, such as survival, proliferation, migration, phagocytosis, autophagy, metabolism, and inflammation. Loss of TREM2 promotes TMEM59 levels, thereby disrupting homeostatic microglial activities.

Herein, we have identified a novel function for TREM2 to regulate TMEM59 protein degradation through protein–protein interactions. Both TREM2 and TMEM59 are type I transmembrane proteins. It has been shown that TREM2 interacts with its ligands through the extracellular N-terminal domain and transduce the signals through the C-terminus that interacts with DAP12. Our results suggested that TREM2 interacted with TMEM59 most likely through their transmembrane domains (Fig. 7). One recent study also found that TMEM59 interacted with the Wnt receptor FZD and LRP6 at the intramembranous domain, and played as a positive regulator of Wnt signaling^49^. Moreover, in addition to previous studies finding that TMEM59 can interact with ATG16L1, an important autophagy factor through a motif near the TMEM59 C-terminus, we showed here that TMEM59 C-terminus could interact with the activated form of LC3B, LC3B-II, and promote autophagic flux. Therefore, different domains of TMEM59 and TREM2 may mediate their diverse functions through interacting with various effectors.

TMEM59 is a ubiquitously expressed protein that also exists in microglia, as well as in neurons and astrocytes. TMEM59 has been shown to modulate APP glycosylation and processing^50^, and regulate autophagy^41,42^. Some recent work also found that TMEM59 could regulate neural stem cell differentiation^43,44^, dendritic spine development^45^, and microglia activation during neuroinflammation^52^. Nevertheless, detailed functions of TMEM59 in the CNS remain largely unclear. Herein, we found that downregulation of TMEM59 reduced pro-inflammatory factor expression and enhanced anti-inflammatory factor expression. Moreover, TMEM59 was downregulated upon inflammatory insults such as LPS treatment. While downregulation of TMEM59 dramatically attenuated LPS treatment-induced inflammatory responses. These results indicate that TMEM59 plays an important role in mediating microglial activities and suggest that TMEM59 reduction may be self-defense of microglia against inflammatory insults.

TREM2 deficiency results in impaired microglial survival, proliferation, migration and phagocytotic activities, and aberrant autophagy and metabolism. We confirmed such impairments and further found that TMEM59 levels were elevated in *Trem2* KO microglia. Importantly, we showed that downregulation of TMEM59 in *Trem2* KO microglia largely reversed these impairments, suggesting that abnormal elevation of TMEM59 is a cause to induce microglial impairments upon loss of TREM2. Since downregulation of TMEM59 in WT microglia also promoted all microglial activities described here, TMEM59 and TREM2 may have parallel and additive functions. In summary, our findings demonstrate the importance of TMEM59 in mediating TREM2 functions in microglia (Fig. 7). Since TREM2 has been considered as a therapeutic target for treating AD, further scrutiny on TMEM59-mediated downstream pathways may provide new avenues for disease intervention.

## Materials and methods

### Cell cultures and animals

HEK293T, Hela, and BV2 cells were originally from ATCC (Manassas, VA, USA) and maintained in our laboratory. These cells and mouse primary microglial cells were prepared and cultured as previously described^19,42,57,58^. *Trem2* KO mice (in C57BL/6N background) and the corresponding WT control mice were from the UC Davis Knockout Mouse Project (KOMP) repository as described previously^59^ and were bred in the Animal Centre of Xiamen University. Animal procedures were in accordance with the National Institutes of Health’s *Guidelines for the Care and Use of Laboratory Animals* and were approved by the Animal Ethics Committee of Xiamen University.

### Plasmids

TMEM59-Myc and TMEM59-GFP were constructed previously^42^. TREM2-Myc, TREM2-GFP, and TREM2-CTF-Myc were also constructed previously^60^. TMEM59-NTF, TMEM59-CTF, and TREM2-NTF were constructed using the pcDNA3.1-Myc/His construct (Thermo Fisher, Waltham, MA, USA) as the backbone. The mCherry-GFP-LC3B plasmid was purchased from BioVector NTCC (Beijing, China). The CSF1R-HA plasmid was purchased from Sino Biological (Beijing, China).

### Transfection

For HEK293T and Hela cells, plasmids were transfected using Turbofect reagent (Thermo Fisher), following the manufacturer’s protocol. For BV2 cells, plasmids were transfected by electroporation using an Amaxa Nucleofector (Lonza, Basel, Switzerland) and Amaxa_Cell Line Nucleofector_Kit T (Lonza), following the manufacturer’s instruction.

### *Tmem59* knockdown by siRNA

Downregulation of *Tmem59* was carried out by electroporation of gene-specific siRNAs using an Amaxa Nucleofector and Amaxa_Cell Line Nucleofector_Kit T (for BV2 cells) or Basic Glial Cells Nucleofector™ Kit (Lonza, for primary microglia), following the manufacturer’s instructions. Each electroporation reaction contained 4 × 10^6^ cells and 200 nM siRNA. The *Tmem59* siRNA used was: 5′-GGACCAAGCUGGAAUGUGATT-3′. A scramble siRNA (5′-UUCUCCGAACGUGUCACGUTT-3′) was used as control. At 48–72 h after electroporation, cells were subjected to further experiments.

### LPS treatment and quantitative real time-PCR (qRT-PCR)

BV2 and mouse primary microglial cells were subjected to *Tmem59* downregulation, and then treated with 500 ng/ml LPS (Sigma-Aldrich, St. Louis, MO, USA) for 6 h. Total RNA was extracted using the TRIzol^®^ reagent (Thermo Fisher) and reverse transcribed into cDNA using the Rever Tra Ace qPCR RT Kit (TOYOBO, Shanghai, China). qRT-PCR was performed using the FastStart Universal SYBR Green Master (Sigma-Aldrich) with primers designed for target genes. PCR primers used were as follows:

β-actin Forward, 5′-AGCCATGTACGTAGCCATCCA-3′;

β-actin Reverse, 5′-TCTCCGGAGTCCATCACAATG-3′;

*Tmem59* Forward, 5′-GGCAGAACTCATCAGGTCGCT-3′;

*Tmem59* Reverse, 5′-GCATTCTTGGCATCAGGGACA-3′;

*Il1β* Forward, 5′-GCAACTGTTCCTGAACTCAACT-3′;

*Il1β* Reverse, 5′-ATCTTTTGGGGTCCGTCAACT-3′;

*Il6* Forward, 5′-CAATGGCAATTCTGATTGTATG-3′;

*Il6* Reverse, 5′-AGGACTCTGGCTTTGTCTTTC-3′;

*Tnfα* Forward, 5′-CCCTCACACTCAGATCATCTTCT-3′;

*Tnfα* Reverse, 5′-GCTACGACGTGGGCTACAG-3′;

*Trem2* Forward, 5′-TGCTGGCAAAGGAAAGGTG-3′;

*Trem2* Reverse, 5′-GTTGAGGGCTTGGGACAGG-3′;

*Arg1* Forward, 5′-CACAGTCTGGCAGTTGGAAGC-3′;

*Arg1* Reverse, 5′-CTTTGGCAGATATGCAGGGAG-3′;

*Ym1* Forward, 5′-CAGGTCTGGCAATTCTTCTGAA-3′;

*Ym1* Reverse, 5′-GTCTTGCTCATGTGTGTAAGTGA-3′.

### Western blot and co-immunoprecipitation

Cells were lysed in NP-40 lysis buffer (25 mM Tris–HCl, pH 7.6, 150 mM NaCl, 1 mM EDTA, and 1% Nonidet P-40) supplemented with the Complete Protease Inhibitor Cocktail (Roche Applied Science, Mannheim, Germany). Equal amounts of protein lysates were analyzed by SDS–polyacrylamide gel electrophoresis, transferred to PVDF membrane (Millipore, Billerica, MA, USA), and immunoblotted with antibodies. Primary antibodies used were: anti-TMEM59 (97597, Abcam, Cambridge, UK); anti-LC3B (3868, Cell Signaling Technology, Danvers, MA, USA); anti-α-tubulin (MABT205, Millipore); anti-Myc (sc-40, Santa Cruz Biotechnology, Dallas, TX, USA); anti-HA (M20003, Abmart, Berkeley Heights, CA, USA); and anti-GFP (M20004, Abmart; or 2956, Cell Signaling Technology). Secondary antibodies used were horseradish peroxidase (HRP)-conjugated goat anti-rabbit IgG (H + L) secondary antibody (31460) and HRP-conjugated goat anti-mouse IgG (H + L) secondary antibody (31430) from Thermo Fisher. Proteins were visualized using ECL Western blotting detection reagents (Millipore). Protein band intensity was quantified using the ImageJ software (National Institutes of Health, Bethesda, MD, USA).

For co-immunoprecipitation, cells were co-transfected with Myc-tagged or GFP-tagged various TREM2 and TMEM59 plasmids for 24 h. Equal amounts of protein lysates were incubated with antibodies against Myc or GFP, or mouse immunoglobulin G (IgG) and Protein G Agarose beads (Thermo Fisher) overnight. Immunocomplexes and 10% amounts of cell lysates for immunoprecipitation (as input) were analyzed by Western blot with antibodies against Myc or GFP to recognize immunoprecipitated proteins.

### Immunofluorescence

Cells were grown on coverslips in 24-well plates. After transfection for 24 h, cells were fixed in 4% paraformaldehyde, permeabilized with 0.2% Triton X-100 in PBS, and blocked in 5% bovine serum albumin. Cells were incubated with an anti-Myc antibody at 4 °C overnight, and incubated with appropriate fluorescence-conjugated secondary antibodies including Alexa-Fluor-594-conjugated goat anti-mouse IgG (A-11005) and Alexa-Fluor-635-conjugated goat anti-mouse IgG (A-31574) from Thermo Fisher. Coverslips were mounted with ProLong^TM^ Gold Antifade Mountant with DAPI (Thermo Fisher). Fluorescence was visualized under a confocal microscope (FV10-ASW, Olympus, Tokyo, Japan).

### Protein degradation assay

BV2 cells were transfected with indicated plasmids and equally split. Cells were then treated with 50 μM CHX (Sigma-Aldrich) for different time periods (0, 2, 4, and 8 h). Alternatively, cells were treated with 50 μM CHX in the presence of 20 μM the proteasomal inhibitor MG132 (ApexBio, Houston, TX, USA) or 30 μM the lysosomal inhibitor NH_4_Cl (Sigma-Aldrich) for 8 h.

### ATP assay

ATP levels in cells were determined based on the phosphorylation of glycerol to generate a product that can be quantified fluorometrically, using an ATP assay kit (ab83355, Abcam) and following the manufacturer’s protocol.

### OCR measurement

The cellular OCR was determined using the Seahorse XF Cell Mito Stress Test Kit (Agilent, Santa Clara, CA, USA) on the Seahorse XFe 96 Extracellular Flux Analyzer (Agilent), following the manufacturer’s protocol. Briefly, 1 × 10^4^ cells per well were seeded into a Seahorse XF 96 cell culture microplate. After baseline measurements, 1 μM oligomycin, 1.5 μM FCCP, and 1.0 μM rotenone–antimycin A were sequentially injected. Data were analyzed using the Seahorse Wave 2.2.0 software package (Agilent). OCR is shown in pmol/min.

### Mitochondrial membrane potential measurement

Mitochondrial membrane potential was assayed using the JC-1 Mitochondrial Membrane Potential Assay Kit (Beyotime, Shanghai, China), following the manufacturer’s protocols. The fluorescence intensities of JC‑1 monomers that dominate in depolarized mitochondria (shown in green) and JC-1 aggregates that dominate in polarized mitochondria (shown in red) were visualized under a confocal microscope (FV10-ASW, Olympus). Fluorescence quantification was performed using ImageJ.

### Growth factor deprivation, ER stress, and cell viability and proliferation assays

Mouse primary microglia were transfected with a scramble control or *Tmem59* siRNA and cultured in media with 30 ng/ml GM-CSF (R&D Systems, Minneapolis, MN, USA) for 24 h, and then in media without GM-CSF for another 48 h. Alternatively, after transfection with a scramble control or *Tmem59* siRNA for 48 h, cells were treated with 10 μg/ml tunicmycin for 24 h to induce ER stress. The viability of treated cells was assessed using a cell counting kit-8 (CCK-8, Dojindo, Rockville, MD, USA), following the manufacturer’s instruction. Cell proliferation was studied using a BrdU cell proliferation ELISA kit (ab126556, Abcam), following the manufacturer’s protocol.

### Transwell migration assay

Cell migration was analyzed using transwell chambers containing 8 µm pore filters (Corning, New York, NY, USA). Briefly, cells were inoculated at a density of 2 × 10^4^ cells/well in the upper chamber with 100 µl DMEM (Hyclone, Logan, UT, USA) supplemented with 10% heat-inactivated FBS (Gibco, New York, NY, USA). The lower chamber was added with 600 µl DMEM supplemented with 10% heat-inactivated FBS. After 48 h of culturing, the media were discarded and chambers were washed twice with serum-free DMEM. Then 100 μl of serum-free DMEM was added into the upper chamber and 600 µl DMEM supplemented with 10% heat-inactivated FBS as chemoattractant was added into the lower chamber. After culturing another 24 h to facilitate cell migration, cells on the upper surface of the insert membrane were fixed in 4% paraformaldehyde and subjected to hematoxylin–eosin staining. Alternatively, cells on the upper surface of the insert membrane were removed using cotton swabs. Migrated cells on the lower side of the filters were fixed and stained with hematoxylin–eosin. Migrated cells in eight randomly selected vision fields under the microscope were photographed; and cell numbers were counted for comparison.

### Phagocytosis assays

Microglial cells were incubated with the pH-dependent pHrodo *E. coli* BioParticles (Thermo Scientific) for 1 h. After washing with PBS, cells were subjected to flow cytometry to measure the fluorescence intensity. Cells that were not incubated with microspheres served as negative controls and were used for background subtraction. Alternatively, after incubating with and removing unbound BioParticles, microglial cells were fixed in 4% paraformaldehyde, and stained with DAPI. The fluorescent signals were captured under a fluorescent microscope (Olympus) and analyzed using ImageJ.

### Statistical analysis

Data were presented as mean ± SEM and analyzed using GraphPad Prism 6 software. Statistical significance was assessed using the Mann–Whitney *U* test between two groups, and using one-way ANOVA with Tukey’s post hoc test when more than two groups were compared. *p* values < 0.05 was considered to be statistically significant.

## Supplementary information

Supplementary Figure legends

Supplementary Figure 1

Supplementary Figure 2

Supplementary Figure 3

Supplementary Figure 4

Supplementary Figure 5

Supplementary Figure 6
