# Supplementary material for: TMEM59 interacts with TREM2 and modulates TREM2-dependent microglial activities
Source: Cell Death Dis. 2020 Aug 13;11(8):678. doi: 10.1038/s41419-020-02874-3 (PMC7442838; doi:10.1038/s41419-020-02874-3)
Supplement: Supplementary file 1 — Supplementary Figure legends [file 41419_2020_2874_MOESM1_ESM.docx]

**Supplementary Figure legends**

**Supplementary Fig. 1 TMEM59 regulates autophagy in HEK293T cells.** HEK293T cells were transfected with control pcDNA or Myc-tagged TMEM59, TMEM59-NTF, and TMEM59-CTF for 24 h. Equal protein amounts of cell lysates were immunoblotted for proteins indicated. Protein levels were quantified by densitometry. The ratio of LC3B-II/LC3B-I was calculated and compared to controls (set to one arbitrary units, AU). Data represent mean ± SEM (n = 3). ns *p*>0.05, ***p*<0.01 (one-way ANOVA with Tukey’s post hoc test).

**Supplementary Fig. 2 Confirmation of TMEM59 downregulation by its siRNA.** Mouse primary microglia were transfected with a *Tmem59* siRNA by electroporation for 0, 12, 24, 36, 48, and 72 h. Equal protein amounts of cell lysates were immunoblotted for proteins indicated. TMEM59 protein levels were quantified by densitometry, normalized to those of α-tubulin, and compared to those at 0 h after transfection (set to one arbitrary units, AU). Data represent mean ± SEM (n = 3). ns *p*>0.05, ***p*<0.01, ****p*<0.001 (one-way ANOVA with Tukey’s post hoc test).

**Supplementary Fig. 3 TMEM59 is involved in inflammation in BV2 cells. a-f** BV2 cells were transfected with a scramble control (SCR) or *Tmem59* siRNA (SI59) for 48 h, and then treated with 500 ng/ml LPS or vehicle control (CON) for 6 h. Gene expression levels of *Tmem59* **(a)**, *Il-1β* **(b)**, *Il-6* **(c)**, *Tnfα* **(d)**, *Arg1* **(e)**, and *Ym1* **(f)** were determined by qRT-PCR and compared to respective controls (set to one arbitrary units, AU). Data represent mean ± SEM (n = 3). ns *p*>0.05, **p*<0.05, ***p*<0.01, ****p*<0.001, *****p*<0.0001 (one-way ANOVA with Tukey’s post hoc test).

**Supplementary Fig. 4 TMEM59 does not interact with CSF1R.** BV2 cells were co-transfected with TMEM59-GFP and CSF1R-HA for 24 h. Cell lysates were immunoprecipitated with antibodies against GFP and HA and mouse immunoglobulin G (IgG), and immunoblotted for the components indicated. Ten percent amounts of cell lysates for immunoprecipitation were used as input.

**Supplementary Fig. 5 TREM2 has no effect on *Tmem59* expression. a,b** BV2 cells were transfected with pcDNA and TREM2-Myc **(a)**, or with pcDNA and TMEM59-Myc **(b)** for 24 h. Gene expression levels of *Tmem59* **(a)** and *Trem2* **(b)** were determined by qRT-PCR and compared to respective controls (set to one arbitrary units, AU). **c** *Tmem59* expression levels in *Trem2* KO mice were determined by qRT-PCR and compared to WT levels (set to one AU). **d** Mouse primary microglia were transfected with a scramble control (SCR) and *Tmem59* siRNA (SI59) for 48 h. *Tmem59* and *Trem2* expression levels were determined and compared to controls (set to one AU). All data represent mean ± SEM (n = 3). **e,f** In BV2 cells, TMEM59-Myc was co-transfected with control (-) or TREM2-NTF-Myc (+) **(e)**, and TREM2-Myc was co-transfected with control (-) or TMEM59-Myc (+) **(f)**. Cells were then equally split, and treated with 50 μM cycloheximide (CHX) for the time indicated. After immunoblotting with an anti-Myc antibody, TMEM59-Myc **(e)** and TREM2-Myc **(f)** protein levels were quantified by densitometry for comparison (values at the 0 h time point were set to one AU). Data represent mean ± SEM (n = 3). **g,h** TMEM59-Myc levels in BV2 cells co-transfected with pcDNA control or TREM2-CTF-Myc at 0 h CHX treatment in Fig. 4e **(g)** and in BV2 cells co-transfected with pcDNA control or TREM2-NTF-Myc at 0 h CHX treatment in **e** **(h)** were quantified and compared to controls (set to one AU). Data represent mean ± SEM (n = 3). **i** HEK293T cells were transfected with TMEM59-Myc or TREM2-Myc. After equally splitting, cells were treated with 50 μM cycloheximide (CHX) for the time points indicated, with or without co-treatment with 20 μM MG132 or 30 μM NH_4_Cl. ns *p*>0.05, ***p*<0.01 (Mann–Whitney U test).

**Supplementary Fig. 6 TMEM59 downregulation promotes survival and proliferation in microglia subjected to growth factor deprivation and ER stress. a,b** Primary microglia derived from wild type (WT) mice were transfected with a scramble control (SCR) or *Tmem59* siRNA (SI59) and cultured in regular media containing 30 ng/ml GM-CSF for 24h. Cells were then cultured in media without GM-CSF (-) for another 48 h. Cell viability was studied using the CCK8 assay **(a)**. Cell proliferation was assessed by BrdU incorporation **(b)**. **c,d** WT mouse primary microglia were transfected with SCR or SI59 for 48 h, and then treated with 10 μg/ml tunicmycin (TUN, +) for another 24 h to induce ER stress. Cell viability was studied using the CCK8 assay **(c)**. Cell proliferation was assessed by BrdU incorporation **(d)**. Data represent mean ± SEM (n = 3). ***p*<0.01 (one-way ANOVA with Tukey’s post hoc test).
