# Supplementary figures and images for: TMEM59 interacts with TREM2 and modulates TREM2-dependent microglial activities
Source: Cell Death Dis. 2020 Aug 13;11(8):678. doi: 10.1038/s41419-020-02874-3 (PMC7442838; doi:10.1038/s41419-020-02874-3)

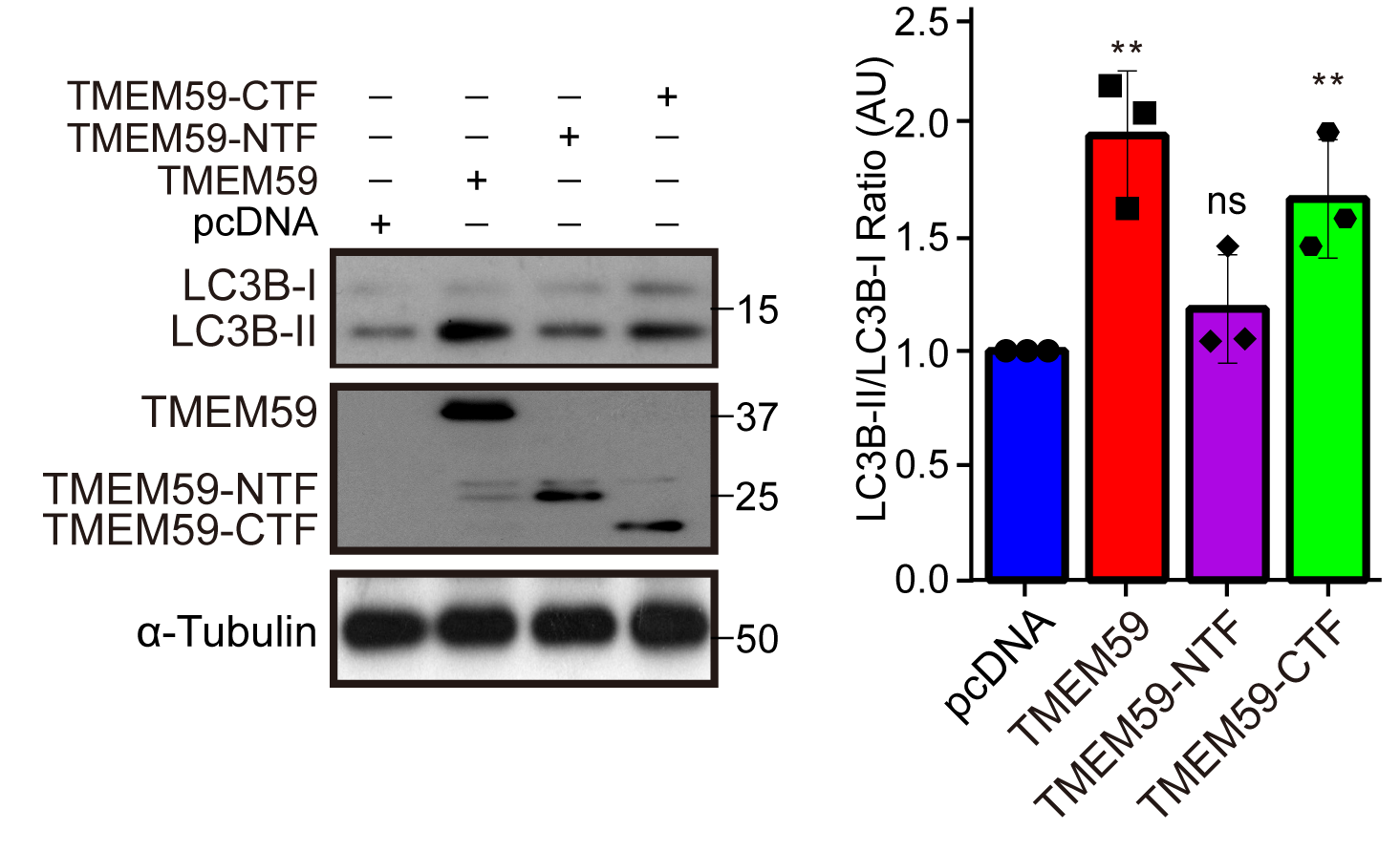

Supplement: Supplementary file 2 — Supplementary Figure 1 [file 41419_2020_2874_MOESM2_ESM.tif]

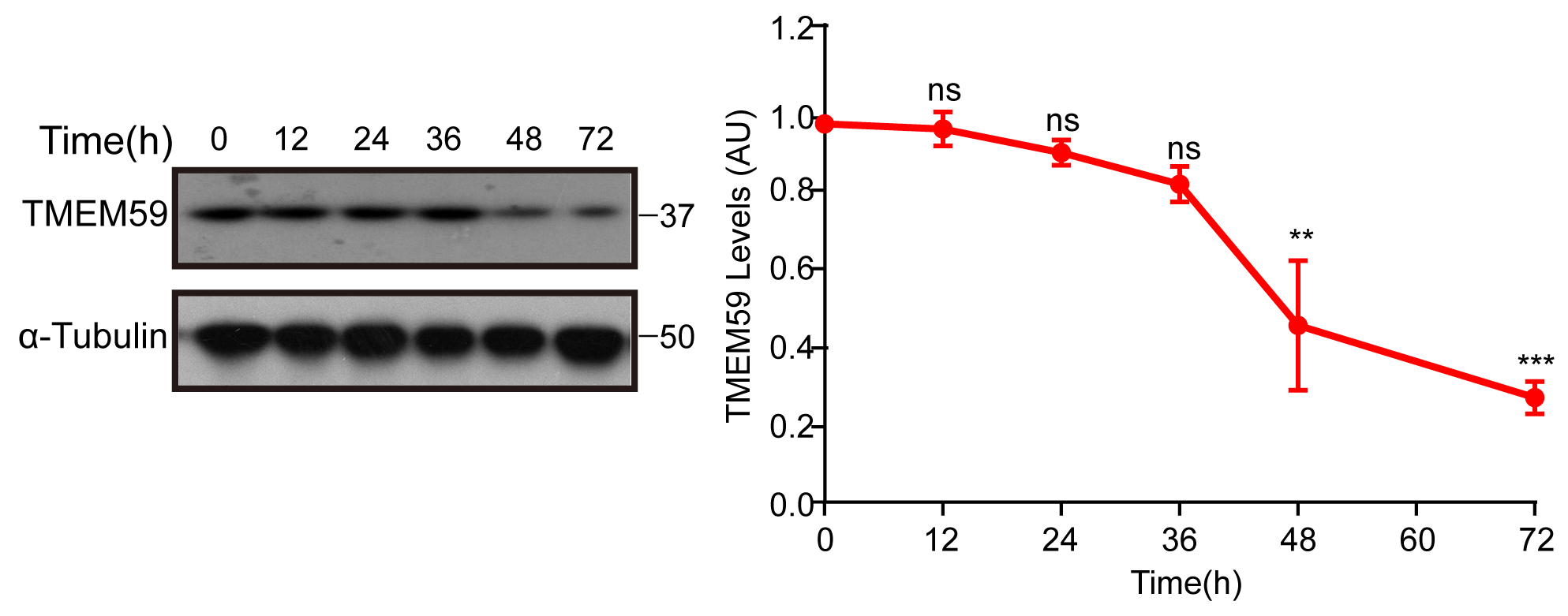

Supplement: Supplementary file 3 — Supplementary Figure 2 [file 41419_2020_2874_MOESM3_ESM.tif]

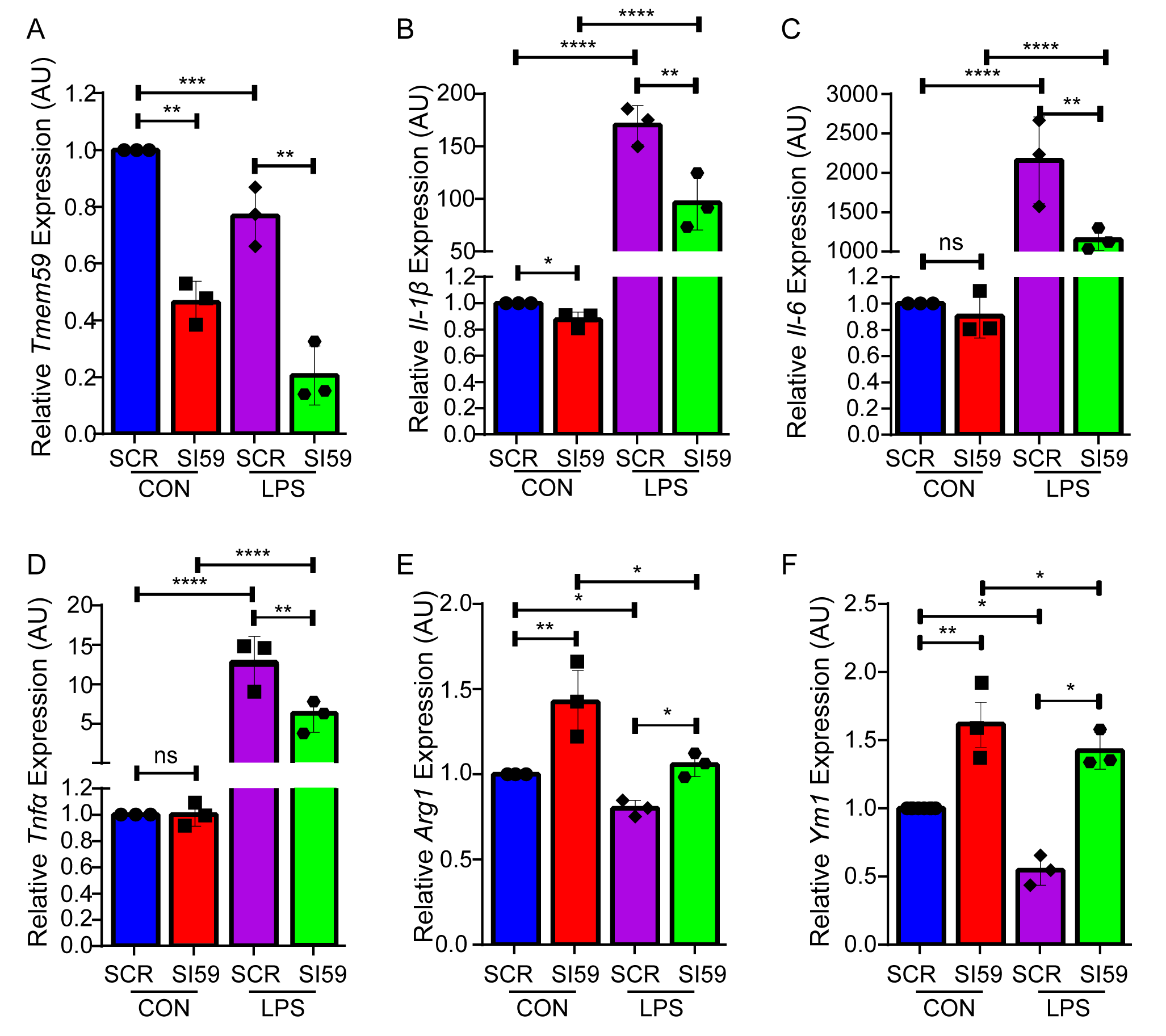

Supplement: Supplementary file 4 — Supplementary Figure 3 [file 41419_2020_2874_MOESM4_ESM.tif]

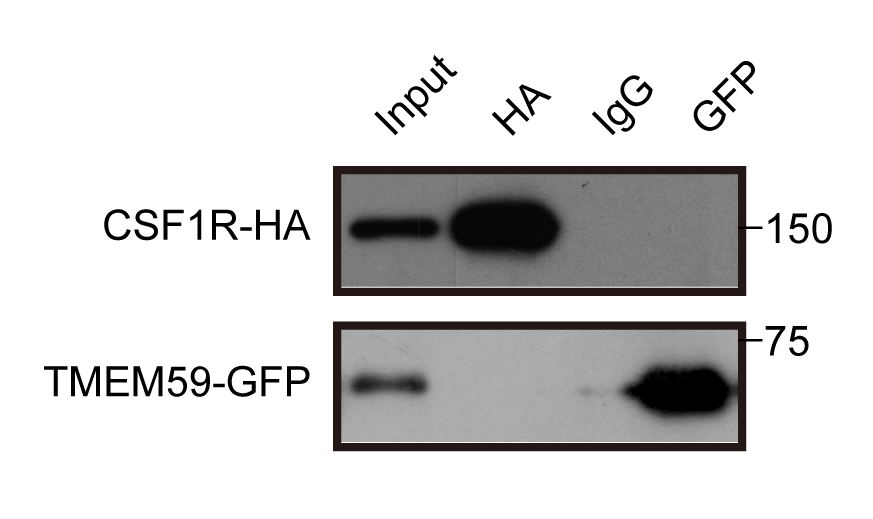

Supplement: Supplementary file 5 — Supplementary Figure 4 [file 41419_2020_2874_MOESM5_ESM.tif]

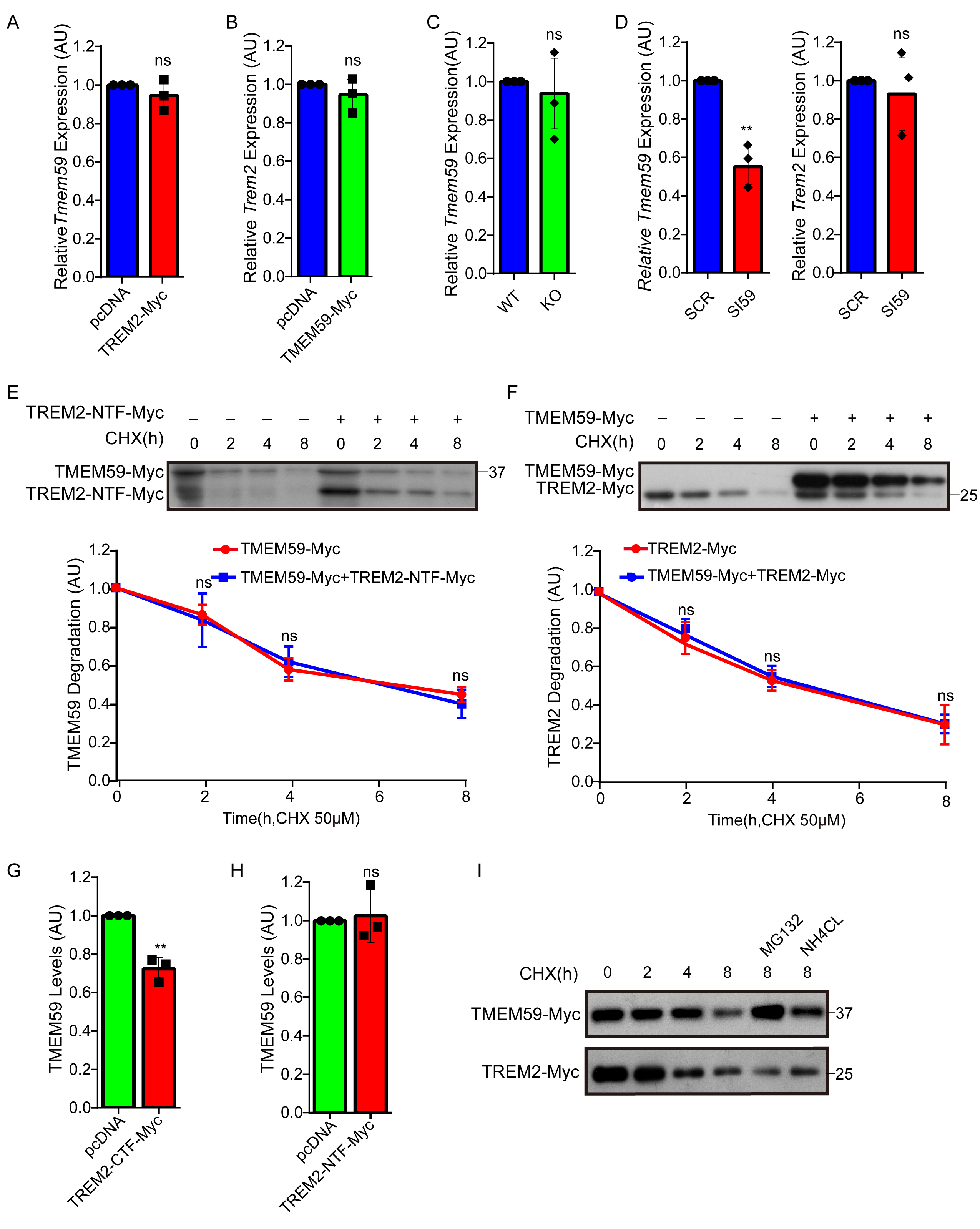

Supplement: Supplementary file 6 — Supplementary Figure 5 [file 41419_2020_2874_MOESM6_ESM.tif]

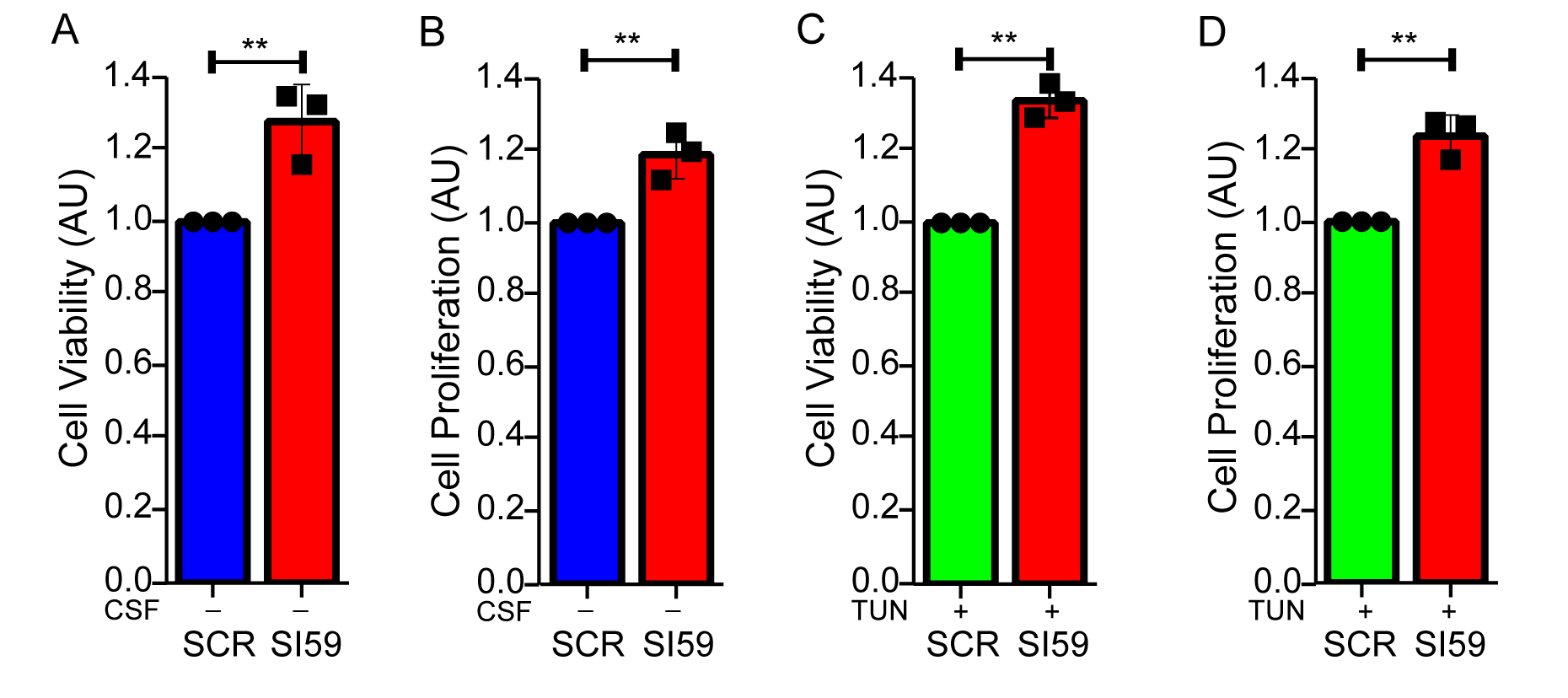

Supplement: Supplementary file 7 — Supplementary Figure 6 [file 41419_2020_2874_MOESM7_ESM.tif]
